# DNA methylation biomarkers for diagnosis of primary liver cancer and distinguishing hepatocellular carcinoma from intrahepatic cholangiocarcinoma

**DOI:** 10.18632/aging.203249

**Published:** 2021-07-08

**Authors:** Yi Bai, Wen Tong, Fucun Xie, Liuyang Zhu, Hao Wu, Rui Shi, Lianjiang Wang, Long Yang, Zhisong Liu, Fei Miao, Qiang Zhao, Yaming Zhang

**Affiliations:** 1 Department of Hepatobiliary Surgery, Tianjin First Central Hospital, School of Medicine, Nankai University, Tianjin, China; 2 Tianjin First Central Hospital Clinic Institute, Tianjin Medical University, Tianjin, China; 3 Department of Liver Surgery, Peking Union Medical College Hospital, Chinese Academy of Medical Sciences and Peking Union Medical College (CAMS and PUMC), Beijing, China; 4 Department of Statistics, Tianjin University of Finance and Economics Pearl River College, Tianjin, China; 5 State Key Laboratory of Medicinal Chemical Biology, Key Laboratory of Bioactive Materials, Ministry of Education, and College of Life Science, Nankai University, Tianjin, China

**Keywords:** methylation, primary liver cancer, hepatocellular carcinoma, intrahepatic cholangiocarcinoma, diagnostic biomarker

## Abstract

Hepatocellular carcinoma (HCC) and intrahepatic cholangiocarcinoma (ICC) are the two most common pathology subtypes of primary liver cancer (PLC). Identifying DNA methylation biomarkers for diagnosis of PLC and further distinguishing HCC from ICC plays a vital role in subsequent treatment options selection. To obtain potential diagnostic DNA methylation sites for PLC, differentially methylated CpG (DMC) sites were first screened by comparing the methylation data between normal liver samples and PLC samples (ICC samples and HCC samples). A random forest algorithm was then used to select specific DMC sites with top Gini value. To avoid overfitting, another cohort was taken as an external validation for evaluating the area under curves (AUCs) of different DMC sites combination. A similar model construction strategy was applied to distinguish HCC from ICC. In addition, we identified DNA Methylation-Driven Genes in HCC and ICC via MethylMix method and performed pathway analysis by utilizing MetaCore. Finally, we not only performed methylator phenotype based on independent prognostic sites but also analyzed the correlations between methylator phenotype and clinical factors in HCC and ICC, respectively. To diagnose PLC, we developed a model based on three PLC-specific methylation sites (cg24035245, cg21072795, and cg00261162), whose sensitivity and specificity achieved 98.8%,94.8% in training set and 97.3%,81% in validation set. Then, to further divide the PLC samples into HCC and ICC, we established another mode through three methylation sites (cg17769836, cg17591574, and cg07823562), HCC accuracy and ICC accuracy achieved 95.8%, 89.8% in the training set and 96.8%,85.4% in the validation set. In HCC, the enrichment pathways were mainly related to protein folding, oxidative stress, and glutathione metabolism. While in ICC, immune response, embryonic hepatocyte maturation were the top pathways. Both in HCC and ICC, methylator phenotype correlated well with overall survival time and clinical factors involved in tumor progression. In summary, our study provides the biomarkers based on methylation sites not only for the diagnosis of PLC but also for distinguishing HCC from ICC.

## INTRODUCTION

Primary liver cancer (PLC) is the sixth commonly diagnosed carcinoma, and it remains the fourth leading cause of cancer-related death. Of note, death from PLC among males is next to lung cancer [1]. The number of PLC patients is increasing worldwide which will lead to a serious health issue and high economic burden. In addition to rare hepatocellular-cholangiocarcinoma (H-ChC), the most common PLC pathological types were hepatocellular carcinoma (HCC) and intrahepatic cholangiocarcinoma (ICC) [2].

The proportion of HCC in PLC was 70%-80%. HCC originates from liver cells. Some risk factors are responsible for its occurrence and development, including hepatitis virus infection, excessive alcohol consumption, autoimmune diseases, and aflatoxin. ICC is the second most common PLC, accounting for 8% - 15% of liver malignant tumors [3]. It originates from intrahepatic bile duct epithelial cells and is mainly associated with biliary tract diseases, for example, sclerosing cholangitis and hepatolithiasis [4].

For some hepatic occupied diseases with atypical imaging and biomarker changes, it is usually difficult to accurately identify the benign and malignant lesions. Because the treatment methods of HCC and ICC are completely different, misdiagnosed may bring disastrous consequences to the patients. Sorafenib was the first systemic therapy approved for the first-line treatment of advanced HCC [5]. While the combination of cisplatin and gemcitabine is the current first-line chemotherapy for patients with advanced-stage cholangiocarcinoma [6]. Accurate diagnosis of PLC type is very important for selecting appropriate treatment methods and making a follow-up schedule. Previous studies have demonstrated that serum biomarkers such as CA19–9 and AFP could be used to differentiate HCC from ICC but the sensitivity and specificity were not satisfied [7]. Therefore, some strategies are urgently needed to improve the certainty and feasibility of diagnosis.

DNA methylation in the promoter CpG island (CGI) of the tumor suppressor gene (TSG), as an important mechanism, usually induces the occurrence and progression of many kinds of cancers [8]. Abnormal methylation of CpG sites in TSGs promoter can change the spatial structure of chromatin, resulting in low or no expression of tumor suppressor genes [9]. Recent studies have shown that abnormal gene methylation is closely correlated with the occurrence of HCC and ICC, which has a potential role in screening the diagnostic biomarkers and therapeutic targets. A previous study demonstrated that circulating tumor DNA methylation markers can be used to distinguish HCC from normal tissues, with a sensitivity of 85.7% and a specificity of 94.3% [10]. Furthermore, DNA methylation of ten CpG sites could be used to distinguish tumor and normal tissue in patients with liver cancer, with a sensitivity of 86% and specificity of 100% [11]. However, these studies only focus on the diagnostic markers of HCC. Few studies are concentrating on the diagnostic markers to distinguish HCC from ICC. In this context, this study aimed to screen methylation biomarkers that could be used to not only confirm the PLC but also distinguish HCC from ICC, which is extremely important for the choice of the subsequent treatment plan.

## RESULTS

### Landscapes of differentially methylated sites in HCC and ICC

**Figure 1:**
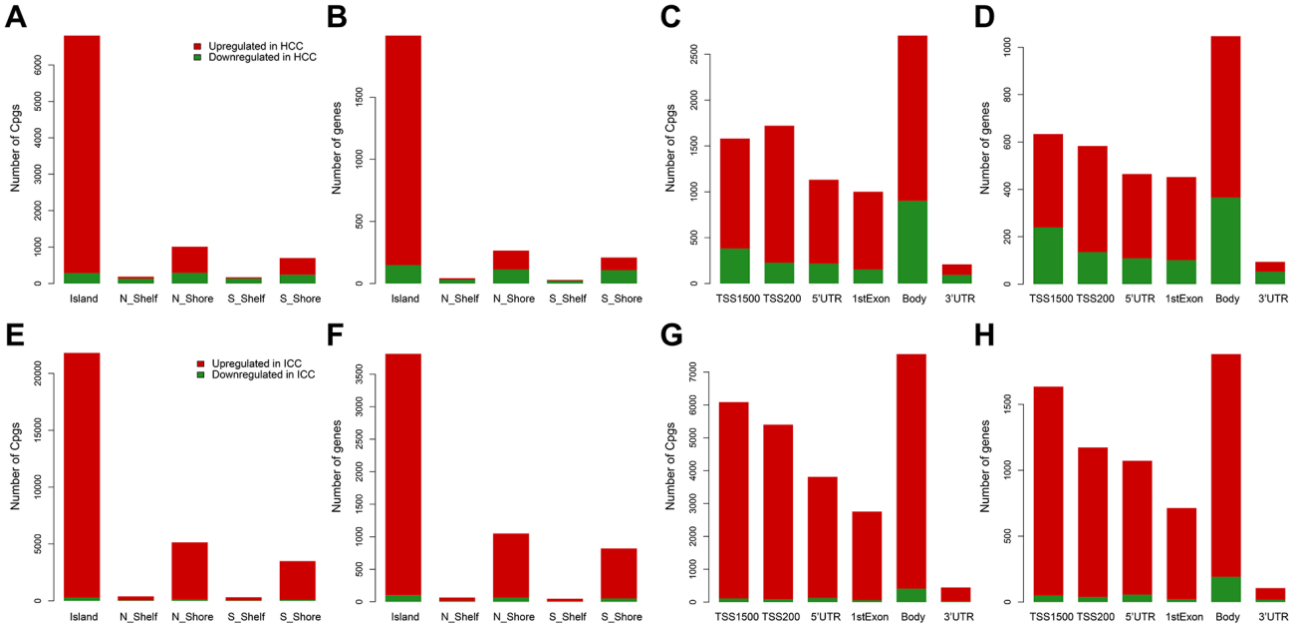
**Distribution of differentially methylated sites and genes in HCC and ICC.** Distribution of differentially methylated sites according to CpG island location in HCC (**A**) and ICC (**E**). Distribution of differentially methylated genes according to CpG island location in HCC (**B**) and ICC (**F**). Distribution of differentially methylated sites according to the distance to the TSS in HCC (**C**) and ICC (**G**). Distribution of differentially methylated genes according to the distance to the TSS in HCC (**D**) and ICC (**H**).

From the training data set in Table 1, methylation data of 96 normal samples and 402 HCC samples were compared. A total of 8,177 hypermethylated sites and 3,152 hypomethylated sites were identified in HCC. While in the comparison of 96 normal samples and 108 ICC samples, there were 33,449 hypermethylated sites and 1,049 hypomethylated sites in ICC. Then according to the genomic region, we visualized the distribution of these DMC sites and corresponding genes. We can see that hypermethylation mainly occurred in CpG islands regardless of HCC (Supplementary Table 1 and Figure 1A, 1B) or ICC (Supplementary Table 1 and Figure 1E, 1F). However, hypomethylation accounted for a higher proportion in the HCC gene body (Supplementary Table 1 and Figure 1C, 1D) compared with ICC (Supplementary Table 1 and Figure 1G, 1H). In promoter regions, both cancer types were dominated by hypermethylation (Supplementary Table 1 and Figure 1C, 1D, 1G, 1H). Such hypermethylation in promoter and hypomethylation in the gene body was considered to be the characteristics of solid tumors. The difference in gene body methylation level also indicates the heterogeneity between HCC and ICC, which proves the feasibility of using DMC sites as potential diagnostic biomarkers.

**Table 1:** The data sets of DNA methylation.

**Training data set**	**Na**	**ICCb**	**HCCc**	**Total**
TCGA-CHOL	8	30	0	38
GSE32079 [20]	0	50	0	50
GSE49656 [21]	4	26	0	30
GSE60753 [22]	34	2	32	68
TCGA-LIHC	50	0	370	420
Total	96	108	402	606
**Validation data set**	**N**	**ICC**	**HCC**	**Total**
GSE89803 [23]	4	96	0	100
DS4-pumch [11]	10	0	10	20
GSE48325 [24]	18	0	0	18
GSE54503 [25]	66	0	66	132
GSE56588 [17]	10	0	224	234
GSE75041	0	0	66	66
GSE77269 [26]	20	0	20	40
GSE89852 [27]	37	0	37	74
GSE99036 [28]	0	0	15	15
GSE107038 [29]	40	0	0	40
GSE113017 [30]	29	0	29	58
GSE113019 [30]	18	0	37	55
Total	252	96	504	852

^a^The number of the normal samples.

^b^The number of the ICC samples.

^c^The number of the HCC samples.

### The sites selection strategy of the diagnostic model

**Figure 2:**
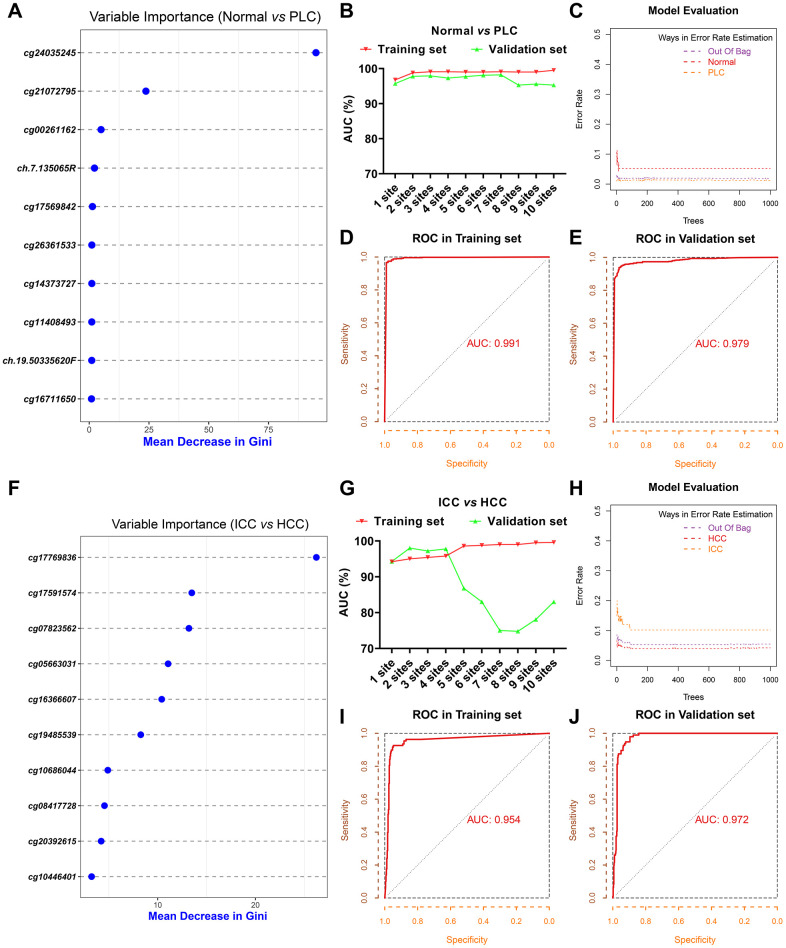
**Screening of diagnostic sites.** The top 10 methylation sites with indicated Gini values for distinguishing PLC and normal (**A**). The AUC curves of the diagnostic prediction model (PLC *versus* Normal) in the training and validation sets based on indicated sites combination (**B**). Evaluation of diagnostic model (PLC *versus* Normal) based on three sites (**C**). The receiver operating characteristic (ROC) curves of diagnostic model (PLC *versus* Normal) based on three sites in the training (**D**) and validation sets (**E**). The top 10 methylation sites with indicated Gini values for distinguishing HCC and ICC (**F**). The AUC curves of the diagnostic prediction model (HCC *versus* ICC) in the training and validation sets based on indicated sites combination (**G**). Evaluation of diagnostic model (HCC *versus* ICC) based on three sites (**H**). The receiver operating characteristic (ROC) curves of diagnostic model (HCC *versus* ICC) based on three sites in the training (**I**) and validation sets (**J**).

To distinguish PLC from benign tumors of the liver, we selected the 6,565 common hypermethylated sites and 187 common hypomethylated sites between HCC and ICC as PLC specific sites pool. Based on the mean decrease of Gini values of methylation sites calculated through the random forest method, we tried different combinations from the top 1 site to the top 10 sites as diagnostic models (Supplementary Table 2 and Figure 2A). As Supplementary Table 3 and Figure 2B shows, the training set AUC values raised as the number of sites increases, while the validation set AUC values declined after 7 sites were included. Hence, to avoid overfitting and ensure the performance of the model, 3 sites (cg24035245, cg21072795, and cg00261162) were selected for economy and simplicity. After model evaluation, the PLC and normal diagnostic error rates are less than 6% (Figure 2C). The AUCs were 0.991 and 0.979 in the training set and validation set, respectively (Figure 2D, 2E).

Next, 11,759 DMC sites between ICC and HCC were used to construct the model for differentiating HCC from ICC followed the same method as described above. The top 10 sites with Gini values were displayed in Supplementary Table 2 and Figure 2F. Although the training set AUC ascended significantly with the augment of the number of sites, the validation set AUC dropped sharply more than 4 sites (Supplementary Table 3 and Figure 2G). The misdiagnosis rate of ICC is about 10%, slightly higher than that of HCC (Figure 2H). The AUC in the training set and validation set based on 3 sites (cg17769836, cg17591574, and cg07823562) are 0.954 and 0.972, respectively (Figure 2I, 2J).

### Performance of the diagnostic model

**Figure 3:**
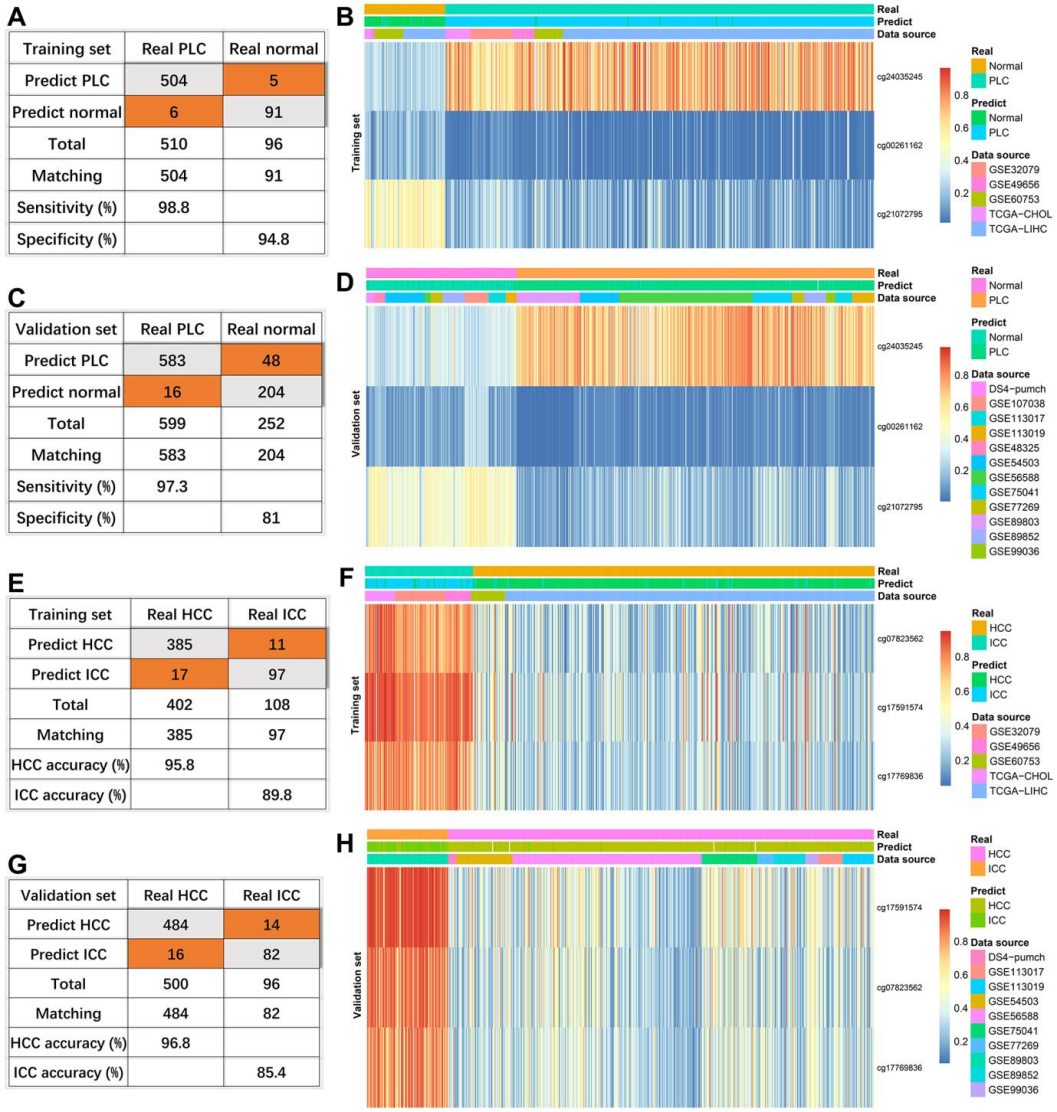
**Effectiveness of diagnostic models.** The diagnosis efficiency of the model for distinguishing between PLC and normal in the training set (**A**) and validation set (**C**). The heatmaps (PLC *versus* Normal) including real status, predict status, data source, and methylation values of indicated sites in the training set (**B**) and validation set (**D**). The diagnosis efficiency of the model for distinguishing between HCC and ICC in the training set (**E**) and validation set (**G**). The heatmaps (HCC *versus* ICC) including real status, predict status, data source, and methylation values of indicated sites in the training set (**F**) and validation set (**H**).

In the training set consisting of 96 normal and 510 PLC, the true positive rate and the true negative rate were 98.8% (504/510) and 94.8% (91/96), respectively (Figure 3A). Because one PLC sample has missing values, 252 normal and 599 PLC were combined as a validation set. The sensitivity and specificity were 97.3% (583/599) and 81% (204/252), respectively (Figure 3C). Compared with the normal group, cg24035245 was hypermethylated, cg21072795 and cg00261162 were hypomethylated in the HCC group regardless of training set or verification set (Figure 3B, 3D).

For three sites discriminating HCC and ICC, four HCC samples with missing values were removed. The diagnostic accuracies of HCC were 95.8% (385/402) and 96.8% (484/500) in the training set and external validation (Figure 3E, 3G). Consistent with the previous results (Figure 2H), the diagnostic accuracies of ICC were lower, 89.8% (97/108) in the training set (Figure 3E) and 85.4% (82/96) in the validation set (Figure 3G). Compared with HCC, the methylation levels of the three sites were higher in ICC in the training set and verification set (Figure 3F, 3H).

Taken together, diagnostic models based on random forest algorithm performed well in distinguishing PLC from normal and distinguishing HCC from ICC.

### DNA methylation-driven genes and related pathways in HCC and ICC

**Figure 4:**
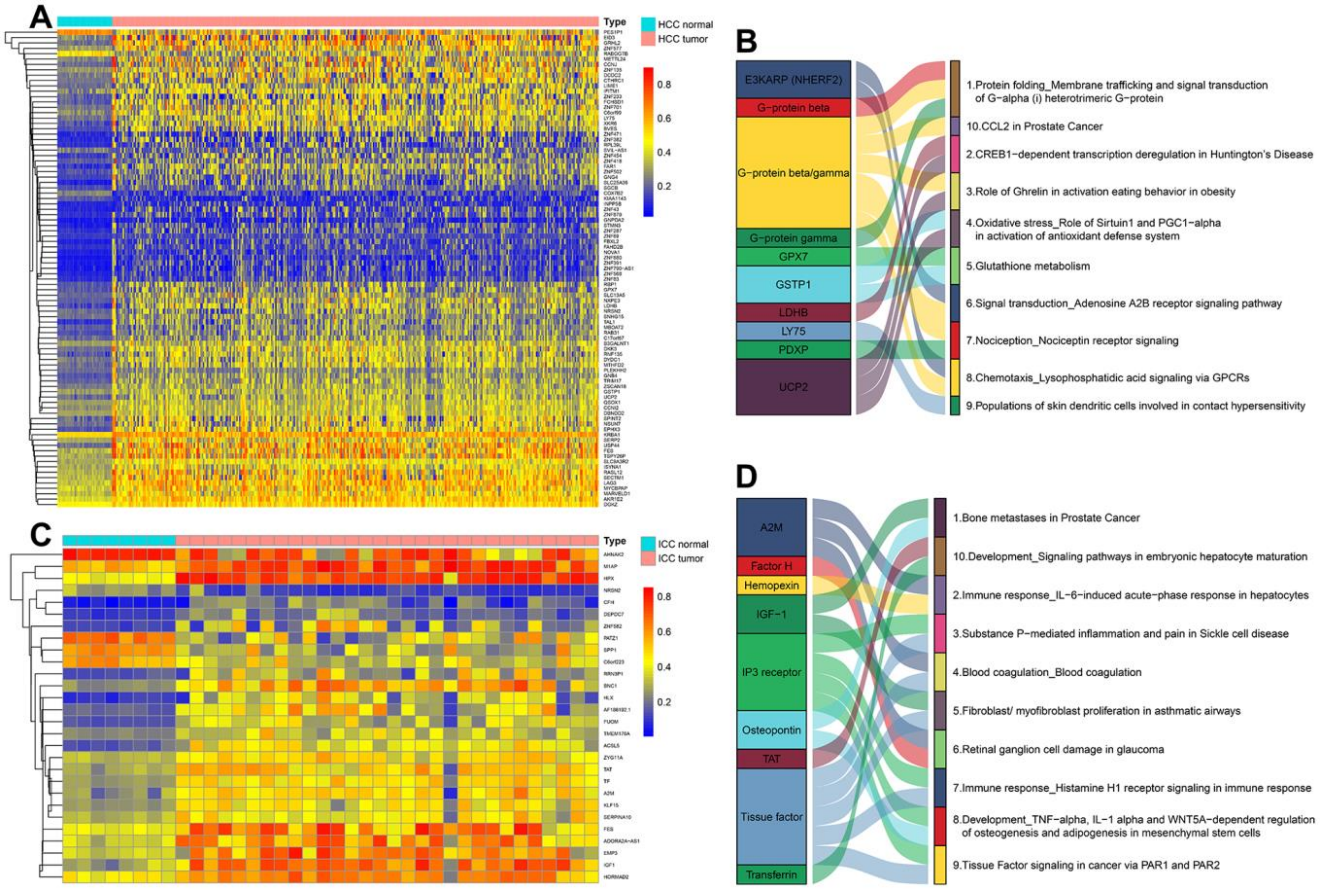
**The methylation heatmaps and enrichment pathways of DNA methylation-driven genes.** The methylation heatmap of DNA Methylation-Driven Genes in HCC (**A**) and ICC (**C**). The enrichment pathways of DNA Methylation-Driven Genes in HCC (**B**) and ICC (**D**).

DNA methylation in the promoter region or near transcription initiation sites always negatively regulated corresponding gene transcription [12]. Among these DMC sites, a total of 89 DNA Methylation-Driven genes (84 hypermethylation genes and 5 hypomethylation genes) were identified in HCC and 28 DNA Methylation-Driven genes (23 hypermethylation genes and 5 hypomethylation genes) in ICC (Figure 4A, 4C and Supplementary Tables 4, 5). The results showed that most of the DNA Methylation-Driven genes were a hypermethylated and down-regulated expression in tumor tissues. To explore the potential function of these genes, pathway enrichment analyses were achieved by MetaCore. In HCC, the enrichment pathways were mainly related to protein folding, oxidative stress, and glutathione metabolism (Figure 4B), which indicates that HCC is a metabolic disease. In ICC, these genes are highly enriched in immune response, embryonic hepatocyte maturation, and tissue factor signaling in cancer via PAR1 and PAR2 (Figure 4D), which suggested the tissue origin of ICC and its close relationship with the immune system.

Taken together, the above results indicate that integrative analysis of promoter DNA methylation and gene expression could facilitate the identification of epigenetic driving factors of cancer.

### Methylator phenotype based on independent prognostic sites in HCC and ICC

**Figure 5:**
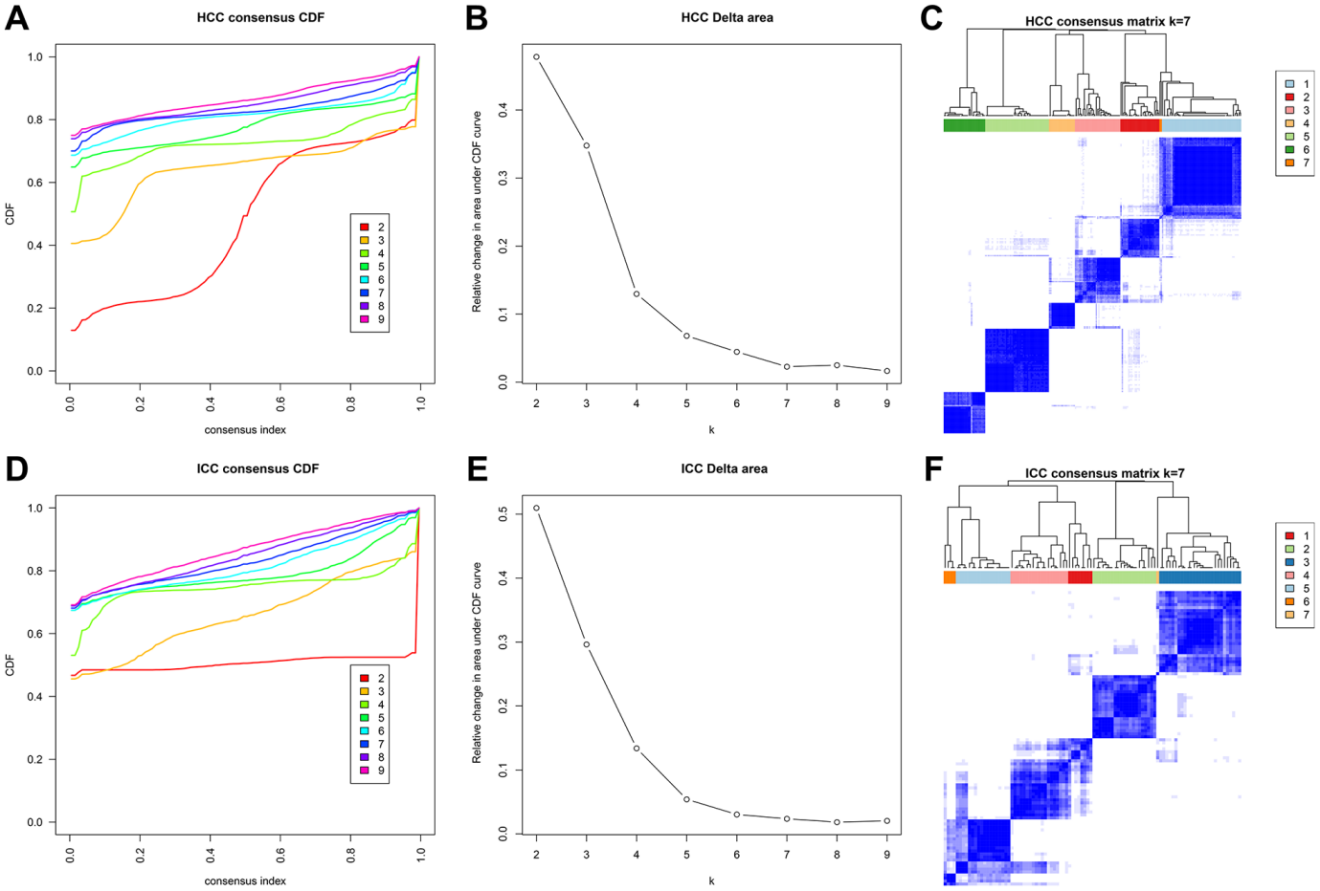
**Methylation typing based on independent prognostic sites.** Consensus cumulative distribution function (CDF) of HCC (**A**) and ICC (**D**). The X-axis represents the consensus index, and the Y-axis represents the CDF curve. The area under the CDF curve for each category in HCC (**B**) and ICC (**E**). The X-axis represents the category number k, and the Y-axis represents the CDF curve. Consensus matrix in HCC (**C**) and ICC (**F**). Different clusters are annotated with indicated numbers and colors. Color gradients in matrix represent consensus values, white corresponds to 0 and dark blue to 1.

Next, 31 independent prognosis associated sites in HCC and 204 independent prognosis associated sites in ICC were identified through univariate and multivariate analyses. Then we performed unsupervised consensus clustering based on these sites. In HCC and ICC, the areas under the CDF curve did not increase significantly after seven categories (Figure 5A, 5B, 5D, 5E). Therefore, HCC and ICC samples were classified into seven clusters. Besides, each cluster has relatively high consistency and low variation (Figure 5C, 5F).

**Figure 6:**
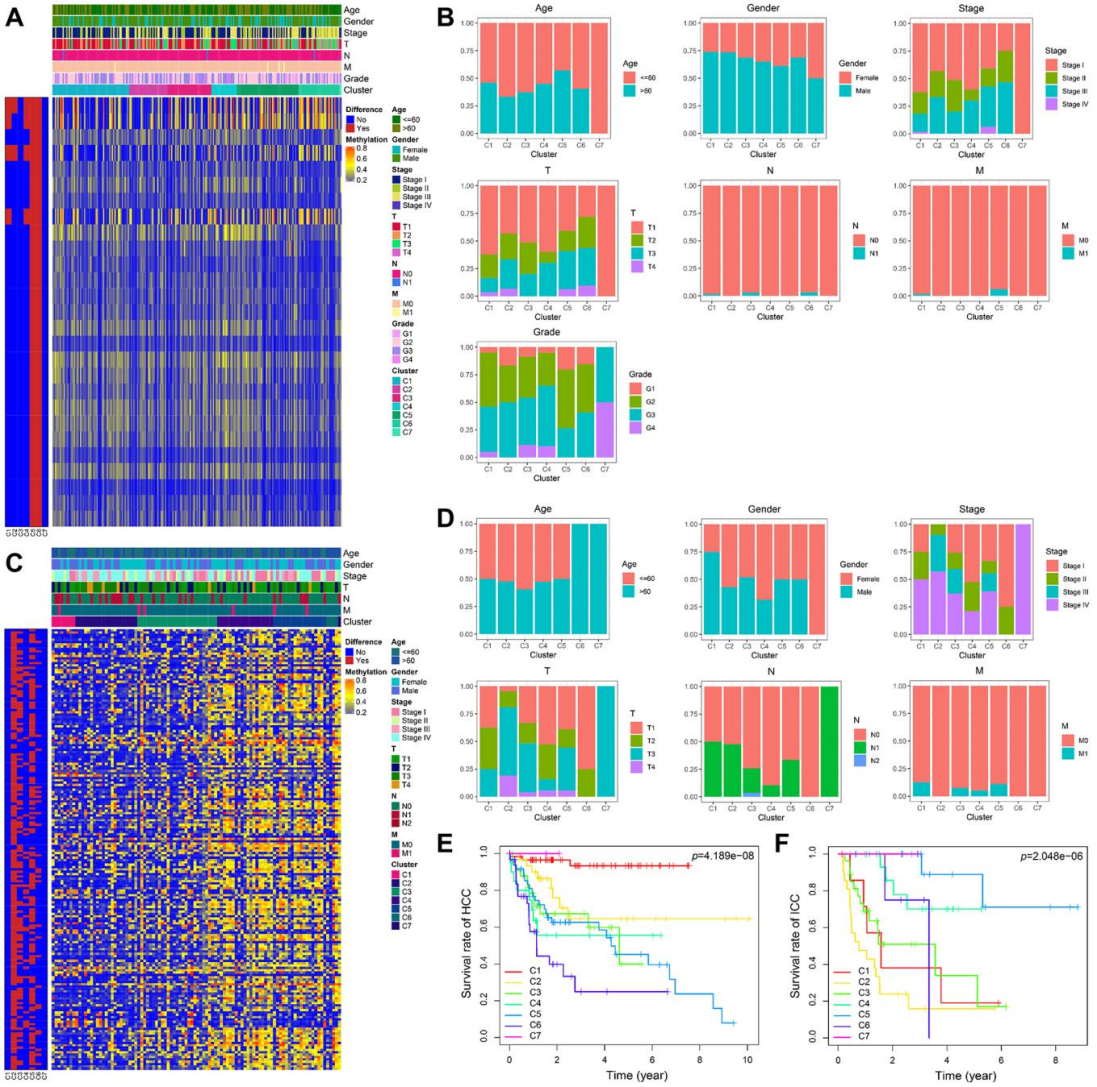
**The methylator phenotype landscape of HCC and ICC.** Heatmaps including DNA methylation classification and clinical factors indicated in HCC (**A**) and ICC (**C**). Comparison of clinical factors indicated among the DNA methylation clusters in HCC (**B**) and ICC (**D**). Kaplan-Meier survival curves of each cluster in HCC (**E**) and ICC (**F**).

The heatmaps annotated with clinical features and DNA methylation subgroups were shown in Figure 6A, 6C. The difference of methylation sites in cluster 5 and 6 was the largest compared with the rest of the samples in HCC. By comparing the clinical characteristics of different clusters, we found that patients in cluster 7 possessed the characteristics of early age (≤60 years), early-stage (stage I), small tumor size (T1), lymph node-negative (N0), no metastasis (M0), and well-differentiated (G3 and G4), which are indicators of better prognosis (Supplementary Table 6 and Figure 6B). Survival analysis also proves this point, cluster 7 has the best prognosis (Figure 6E). We can see that stage II and III accounted for the largest proportion in cluster 6 with the worst prognosis (Figure 6B, 6E). It suggested that patients in the later stage always have a shorter overall survival time.

In ICC, the methylation profiles of cluster 2 and cluster 5 have significant differences with other samples (Figure 6C). However, their prognosis was the opposite (Figure 6F). Compared with patients in cluster 5, more advanced stage and tumor invasive extent were observed in cluster 2 with the worst prognosis (Supplementary Table 7 and Figure 6D).

To sum up, the dysregulated methylation intensity can not only be used as prognostic biomarkers but also correlated well with clinical factors involved in tumor progression.

## DISCUSSION

The liver is the most common site of the tumor, and also the easily metastatic site of a malignant tumor from other organs [13]. HCC and ICC are important components of PLC. They have similar clinical symptoms and imaging findings, such as a round mass in the liver, abdominal pain, and abdominal distension, etc. [14, 15]. But in terms of etiology, pathogenesis, and treatment, ICC is different from HCC. Most patients with ICC are advanced at the time of diagnosis, it always hard to radical resect these no complete capsule tumors infiltrating the surrounding organs. Hence, the development of useful diagnostic biomarkers will contribute to timely and effective treatment, which may improve the prognosis of patients.

The CpG methylation site is one of the most powerful biomarkers in cancer. Accumulated studies have shown that the occurrence and development of HCC and ICC were associated with promoter hypermethylation [16]. Dysregulated DNA methylation occurs in the early stage of cancer, even though the tissue at this time was pathologically diagnosed as normal [17]. The major changes in tumors were composed of global changes and local changes at the site level (especially hypermethylation of CpG island and promoter) [18]. The whole-genome DNA hypomethylation (GDH) and the CpG island methylator phenotype (CIMP) were identified at about 90% tumor samples [11]. Therefore, epigenetic changes can be detected alone or combined with other biomarkers for accurate diagnosis of HCC and ICC.

Through differential methylation site analysis, we found that promoter regions were the most hypermethylated in tumors. The dysfunction of tumor suppressor genes is associated with aberrant methylation in the promoter region, which leads to tumorigenesis. It was reported that in tumor hypomethylation mainly occurs in the gene body region, while hypermethylation in the promoter region [19]. This is consistent with our findings. Besides, HCC and ICC have their specific methylation changes.

The existing research mainly focused on diagnostic biomarkers of HCC or ICC, and few studies have been conducted on biomarkers used to distinguish HCC from ICC. The key finding of this research is the identification of several specific methylated sites as potential diagnostic biomarkers for not only distinguishing PLC from normal but also HCC from ICC. Random forest is an ensemble learning method, which has extremely high accuracy, can effectively run-on large data sets. The contribution of sites can be reflected by Gini values. However, how many sites are included to ensure the accuracy and universality of the model has become a new challenge. Here we proposed an approach to prevent the overfitting of models. The AUCs of the models will expand with the increase of the included variables in the training sets of two diagnostic models. We also calculated the AUCs of different site combinations in the validation set. As expected, when the AUCs reach the peak, it will decrease with the increase of the included sites. To reduce the overfitting, we chose the least combination of sites that could reach the peak of AUC for its cost-effectiveness. We first build a PLC diagnostic model including cg24035245, cg21072795, and cg00261162 to distinguish PLC from normal. If it is PLC, we further differentiate between HCC and ICC through another three sites (cg17769836, cg17591574, and cg07823562). By detecting these six methylated sites, we can identify the pathological subtypes of PLC with high sensitivity and specificity.

Additionally, we identified some DNA Methylation-Driven Genes in HCC and ICC by integrated analyzing DNA methylation and gene expression data. Hypermethylation genes account for high proportions in both cancer types. In HCC, DNA Methylation-Driven Genes are mainly involved in metabolic-related signaling pathways. While in ICC, they mainly regulate the origin and immune microenvironment of cholangiocarcinoma.

Last, we used independent prognostic sites to classify HCC and ICC. After survival and clinical correlation analyses, we found later stages correlated well with methylation sites, which is a biomarker for bad prognosis in both tumor subtypes. Of note, ICC infiltration degree is a poor prognostic factor for ICC, which should be paid more attention to in clinical practice.

There are also some limitations in our study. Because biopsy is still traumatic, the diagnostic efficacy of these biomarkers should be further verified in peripheral blood. In the following study, we will develop a simple and sensitive technique to measure the methylation level of cell-free ctDNA by extracting peripheral blood. By detecting the methylation level of ctDNA, we can compare the uniformity of methylation level in tumor tissue and blood and validate the diagnosis efficiency.

## CONCLUSIONS

Our study established a two-step diagnosis model based on differentially methylated sites. Firstly, cg24035245, cg21072795, and cg00261162 were used to diagnosis PLC. If the diagnosis of PLC was considered, cg17769836, cg17591574, and cg07823562 were used to further distinguish HCC from ICC. Additionally, we identified DNA Methylation-Driven Genes related pathways and performed methylator phenotype based on independent prognostic sites in HCC and ICC, respectively.

## MATERIALS AND METHODS

### Identification of differentially methylated CpG sites

HCC and ICC related DNA methylation array data sets of Table 1 detected by Illumina Human Methylation450 BeadChip (GPL13534) were download from UCSC Cancer Browser (https://xenabrowser.net/datapages/) and the Gene Expression Omnibus (GEO) database (https://www.ncbi.nlm.nih.gov/geo/). Probe removal criteria were as follows: (1) The missing β value of methylation site >30%; (2) Methylation sites in the sex chromosome. Only shared methylation sites in training data sets were retained and performed Wilcoxon rank-sum tests in three groups (96 normal samples *versus* 402 HCC samples, 96 normal samples *versus* 108 ICC samples, and 108 ICC samples *versus* 402 HCC samples) after replenishing residual missing values with the Bioconductor package impute. Sites with an adjusted *P*-value < 0.05 and a |log2FoldChange| > 1 (log2FC) were considered DMC sites unless noted elsewhere.

### Construction and optimization of diagnosis model

The diagnosis process is divided into two steps. The first step is the diagnosis of PLC. The second step is to further distinguish between HCC and ICC. Random forest method was utilized to select candidate sites with high Gini values from DMC sites (Normal *versus* PLC: common DMC sites of 96 normal samples *versus* 108 ICC samples and 96 normal samples *versus* 402 HCC samples; ICC *versus* HCC: 108 ICC samples *versus* 402 HCC samples) [31]. Then the combination of diagnostic sites with the minimum number and the highest AUC in both training and validation data sets (Table 1) were confirmed to avoid overfitting.

### Pathway analysis of DNA methylation-driven genes

DNA methylation data and RNA-seq counts of TCGA-LIHC (41 normal and 364 HCC) and TCGA-CHOL (8 normal and 30 ICC) were acquired from The Cancer Genome Atlas (TCGA) portal (https://portal.gdc.cancer.gov/). The mean value of all methylation sites in promoter regions (from -1500 to +500 of the transcription start sites) was considered as the methylation value of the gene. The gene expression data were normalized via the edgeR method [32]. The DMC sites between normal and HCC or ICC were annotated as genes (If a site matched multiple genes, the first one was chosen as a reference). Then Bioconductor package MethylMix was used to screen DNA Methylation-Driven Genes (The correlation coefficient between selected gene methylation value and gene expression < -0.3 and an adjusted *P*-value < 0.05) [33]. Enrichment pathway maps were achieved from MetaCore (https://portal.genego.com/). The mean values of normal samples and tumor samples of gene expression normalized by edgeR were used as input files and the top 10 enrichment pathways were illustrated by the Sankey diagram.

### Methylator phenotype of prognostic sites

Independent prognostic related HCC DMC sites were screened after univariate and multivariate analyses in TCGA LIHC (360 samples with survival time, 229 samples with complete clinical data). Similar in ICC, TCGA-CHOL (30 samples with survival time, 24 samples with complete clinical data) and GSE89803 (94 samples with survival time, 74 samples with complete clinical data) were combined for identifying prognostic sites. Based on their respective sites, the R package ConsensusClusterPlus was used to perform K-means-based consensus clustering [34]. The overall survival rates were estimated through the Kaplan-Meier approach.

## Supplementary Material

Supplementary Table 1

Supplementary Tables 2 and 3

Supplementary Table 4

Supplementary Table 5

Supplementary Table 6

Supplementary Table 7
