# Supplementary material for: DNA methylation biomarkers for diagnosis of primary liver cancer and distinguishing hepatocellular carcinoma from intrahepatic cholangiocarcinoma
Source: Aging (Albany NY). 2021 Jul 8;13(13):17592–606. doi: 10.18632/aging.203249 (PMC8312421; doi:10.18632/aging.203249)
Supplement: Supplementary Tables 2 and 3 [file aging-13-203249-s002.pdf]

## SUPPLEMENTARY TABLES

**Supplementary Table 2. The top 10 sites with Gini values.**

| Variable importance (Normal *vs* PLC) | |
|---|---|
| **Name** | **Mean decrease in Gini** |
| cg24035245 | 94.87351299 |
| cg21072795 | 23.72076107 |
| cg00261162 | 4.949928302 |
| ch.7.135065R | 2.198442961 |
| cg17569842 | 1.361948487 |
| cg26361533 | 1.099281746 |
| cg14373727 | 1.097849508 |
| cg11408493 | 1.081394428 |
| ch.19.50335620F | 1.059194795 |
| cg16711650 | 0.956828283 |
| **Variable importance (ICC *vs* HCC)** | |
| **Name** | **Mean decrease in Gini** |
| cg17769836 | 26.23050017 |
| cg17591574 | 13.48966713 |
| cg07823562 | 13.19458909 |
| cg05663031 | 11.06424738 |
| cg16366607 | 10.41241696 |
| cg19485539 | 8.265638085 |
| cg10686044 | 4.8977075 |
| cg08417728 | 4.555597011 |
| cg20392615 | 4.218905655 |
| cg10446401 | 3.238699251 |

Relating to Figure 2A, 2F.

**Supplementary Table 3. AUC of models with different number of sites.**

| **Normal *vs* PLC** | **1 site** | **2 sites** | **3 sites** | **4 sites** | **5 sites** | **6 sites** | **7 sites** | **8 sites** | **9 sites** | **10 sites** |
|---|---|---|---|---|---|---|---|---|---|---|
| Training set | 0.968 | 0.988 | 0.991 | 0.991 | 0.99 | 0.99 | 0.991 | 0.99 | 0.99 | 0.995 |
| Validation set | 0.957 | 0.978 | 0.979 | 0.973 | 0.977 | 0.981 | 0.982 | 0.953 | 0.956 | 0.953 |
| ICC *vs* HCC | 1 site | 2 sites | 3 sites | 4 sites | 5 sites | 6 sites | 7 sites | 8 sites | 9 sites | 10 sites |
| Training set | 0.942 | 0.95 | 0.954 | 0.958 | 0.986 | 0.988 | 0.99 | 0.99 | 0.995 | 0.996 |
| Validation set | 0.943 | 0.98 | 0.972 | 0.978 | 0.868 | 0.83 | 0.75 | 0.748 | 0.781 | 0.83 |

Relating to Figure 2G.
