# Supplementary material for: DNA methylation biomarkers for diagnosis of primary liver cancer and distinguishing hepatocellular carcinoma from intrahepatic cholangiocarcinoma
Source: Aging (Albany NY). 2021 Jul 8;13(13):17592–606. doi: 10.18632/aging.203249 (PMC8312421; doi:10.18632/aging.203249)
Supplement: Supplementary Table 5 [file aging-13-203249-s004.pdf]

SUPPLEMENTARY TABLE

Supplementary Table 5. Results of differential analysis between normal samples and ICC samples.

Gene	Normal mean	ICC mean	logFC	P value	Adjust P	cor	cor P value
NRSN2	0.265258974	0.12171362	-1.12391	3.93E-06	0.000573	-0.60727	0.000373
PATZ1	0.65938025	0.34791105	-0.92239	1.64E-07	2.39E-05	-0.60113	0.000443
SPP1	0.605498782	0.3271744	-0.88806	0.000183	0.026664	-0.59963	0.000462
C6orf223	0.572928919	0.35862386	-0.67588	3.93E-06	0.000573	-0.58952	0.000608
AHNAK2	0.790859731	0.58680545	-0.43054	0.000202	0.029526	-0.70736	1.24E-05
M1AP	0.538616569	0.72546894	0.429655	2.74E-06	0.0004	-0.62173	0.000245
HORMAD2	0.438715914	0.60332508	0.459649	0.000137	0.020002	-0.82221	2.51E-08
SERPINA10	0.306045445	0.42803955	0.483998	0.000107	0.015668	-0.6095	0.00035
ZYG11A	0.316567749	0.46899738	0.567066	0.000182	0.026642	-0.57065	0.000992
FES	0.400817591	0.60842559	0.602135	1.89E-05	0.002765	-0.78972	2.12E-07
TMEM176A	0.228656995	0.34725144	0.602796	2.74E-06	0.0004	-0.62337	0.000233
ADORA2A-AS1	0.349828035	0.57527269	0.7176	7.69E-06	0.001123	-0.58009	0.000779
EMP3	0.354403029	0.58503871	0.723141	0.000202	0.029526	-0.6061	0.000385
KLF15	0.258977664	0.43466174	0.747065	5.37E-05	0.00784	-0.65116	9.74E-05
TAT	0.293132521	0.49681662	0.76116	5.56E-06	0.000812	-0.63698	0.000154
HPX	0.416278862	0.73742367	0.824943	1.84E-06	0.000269	-0.71835	7.82E-06
TF	0.263338122	0.46728678	0.827392	2.74E-06	0.0004	-0.62542	0.000219
A2M	0.249939542	0.4816613	0.94644	2.74E-06	0.0004	-0.6927	2.22E-05
DEPDC7	0.104488228	0.2085257	0.996885	1.06E-05	0.001541	-0.59664	0.000501
RRN3P1	0.147728654	0.32820824	1.151662	3.25E-05	0.004747	-0.68214	3.30E-05
IGF1	0.286284071	0.64556513	1.173115	1.84E-06	0.000269	-0.71957	7.42E-06
BNC1	0.24159043	0.54522145	1.174279	2.74E-06	0.0004	-0.59823	0.00048
FUOM	0.150583042	0.34631798	1.201538	2.74E-06	0.0004	-0.5788	0.000806
AF186192.1	0.173482553	0.40342318	1.217503	3.25E-05	0.004747	-0.60556	0.000391
ACSL5	0.161039038	0.40662332	1.336282	4.09E-08	5.97E-06	-0.60973	0.000348
ZNF582	0.124543989	0.32179253	1.369475	0.000133	0.019477	-0.65413	8.83E-05
HLX	0.106896714	0.33317347	1.640056	1.06E-05	0.001541	-0.60329	0.000417
CFH	0.04875832	0.19137859	1.972709	0.000247	0.036035	-0.58316	0.000719

Relating to Figure 4C.
